# Unforeseen Sequelae: Myxomatous Aneurysm and Cerebral Metastasis in a Case of Atrial Myxoma—A Clinical Image 

**DOI:** 10.31083/RN37950

**Published:** 2025-02-12

**Authors:** Sukalyan Purkayastha, Rajinder Kumar, Dinesh Verma, Deepak Dhurvey, Nitin Kumar, Surajit Jana

**Affiliations:** ^1^Department of Neurointervention, Institute of Neurosciences Kolkata, 700017 Elgin, Kolkata, India

Cardiac myxomas, often found in the left atrium, are benign tumors but can have malignant behavior by causing life-threatening embolic events, notably affecting the central nervous system [1]. Such embolizations can lead to ischemic strokes and, rarely, myxomatous aneurysms and brain metastases [1]. Myxomatous aneurysms, typically fusiform and located in distal cortical vessels, form when myxoma emboli infiltrate and damage the vessel wall, resulting in pseudoaneurysm formation [2, 3]. True parenchymal metastases, resulting from vessel wall transgressions, are exceedingly rare and usually occur adjacent to these aneurysms [3].

For multiple aneurysms or cerebral metastases, chemotherapy with agents like doxorubicin or ifosfamide, potentially alongside whole-brain radiotherapy, may be considered to extend recurrence-free intervals [3, 4]. Due to the rarity of myxomatous aneurysms and parenchymal metastases, standardized treatment protocols are lacking [3, 4]. Management typically involves the use of anticoagulants or antiplatelet agents to prevent further embolic events [3, 4]. For cases with a few isolated aneurysms or when lesions pose a life-threatening risk, surgical or endovascular interventions may be appropriate [2, 3, 4]. For multiple aneurysms or cerebral metastases, chemotherapy with agents like doxorubicin or ifosfamide, potentially alongside whole-brain radiotherapy, may be considered to extend recurrence-free intervals [3, 4].

We encountered a 42-year-old male, with a history of total excision of a left atrial myxoma in April 2023, presented in September 2024 with mild weakness, tingling, and numbness in the left upper and lower limbs for two months. He also experienced persistent headaches and had two episodes of generalized tonic-clonic seizures. An echocardiogram confirmed no recurrence of the atrial myxoma. Brain magnetic resonance imaging (MRI) revealed T2/fluid attenuated inversion recovery sequences (FLAIR) heterogeneous mixed-signal intensity lesions in the bilateral parietal, right temporal-occipital, and right frontal regions, accompanied by surrounding edema and heterogeneous post-contrast enhancement, suggestive of parenchymal metastasis (Fig. 1). Digital subtraction angiography (DSA) of the brain demonstrated multiple fusiform aneurysms in the cortical branches of the bilateral anterior cerebral artery (ACA), middle cerebral artery (MCA), and posterior cerebral artery (PCA) (Fig. 2). The treatment options were discussed with him, and he opted to undergo chemotherapy and radiotherapy.

**Fig. 1.**
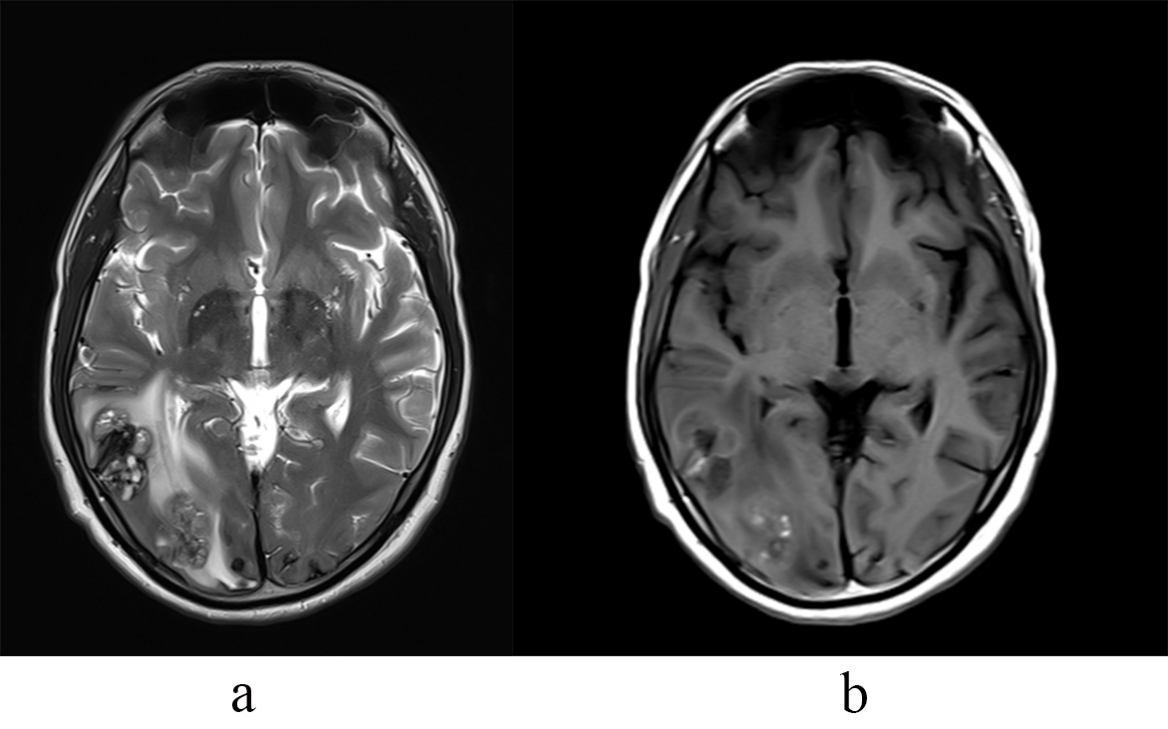
**Right temporo-occipital lesions on MRI**. (a) T2W MRI shows right temporo-occipital lesions with heterogeneous signal and edema. (b) T1 Post contrast shows mild heterogeneous enhancement. T2W MRI, T2 weighted magnetic resonance imaging.

**Fig. 2.**
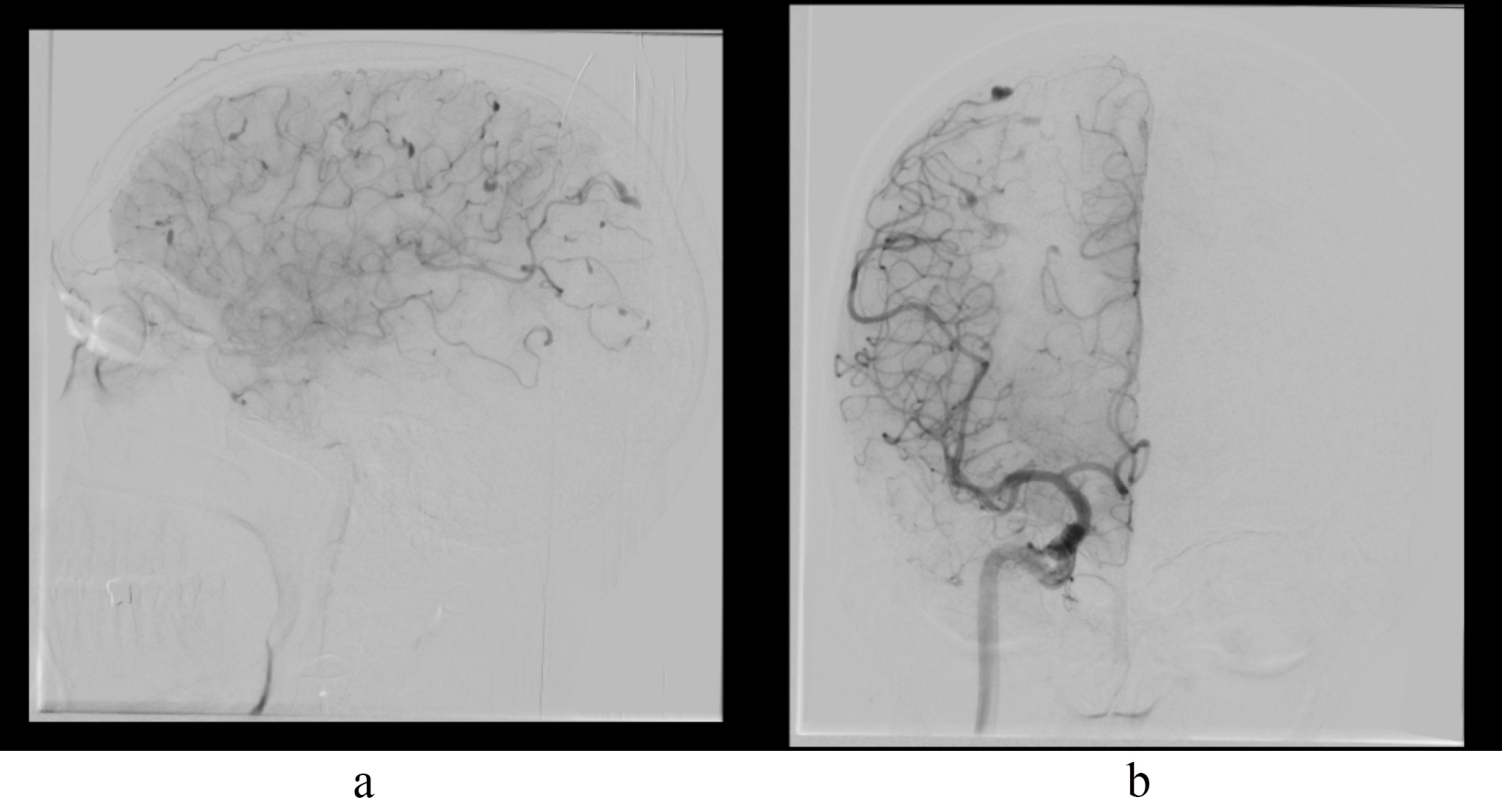
**Multiple fusiform aneurysms on DSA**. (a) DSA lateral view shows multiple fusiform aneurysms in ACA and MCA cortical branches. (b) DSA AP view shows multiple fusiform aneurysms in ACA and MCA cortical branches. DSA, digital subtraction angiography; ACA, anterior cerebral artery; MCA, middle cerebral artery; AP, antero-posterior view.

## Data Availability

All data and materials are included in the article and additional information is available from the corresponding author upon request.
